# Experimental Characterization and Non-Linear Dynamic Modelling of PCD Bearings: A Digital-Twin Approach for the Condition Monitoring of Rotating Machinery

**DOI:** 10.3390/s26082545

**Published:** 2026-04-20

**Authors:** Alessio Cascino, Andrea Amedei, Enrico Meli, Andrea Rindi

**Affiliations:** Department of Industrial Engineering, University of Florence, 50139 Florence, Italy

**Keywords:** PCD bearings, turbomachinery, multibody dynamic modelling, experimental modal validation, subsea machinery monitoring, rotor balancing, non-linear contact forces

## Abstract

This study proposes a comprehensive methodology for the experimental characterization and non-linear dynamic modelling of Polycrystalline Diamond (PCD) bearings, establishing a high-fidelity digital twin approach for the condition monitoring of rotating machinery. The research addresses complex rotor–stator interactions through the development of a multibody numerical framework. A structural 1D Finite Element (FE) model of the stator assembly was first calibrated via experimental modal analysis, achieving a high correlation with the first four bending modes and a maximum frequency discrepancy of only 1.4%. This validated structure was integrated into a non-linear multibody environment to simulate transient rub-impact events at rotational speeds up to 5500 rpm across varying clearance configurations. The model successfully captures the transition from stable periodic orbital motion to the stochastic and chaotic regimes observed in high-clearance setups. Frequency-domain validation further confirms the model’s accuracy in identifying supersynchronous harmonics and energy distribution patterns. Quantitative analysis shows that high-clearance configurations generate impact forces exceeding 6000 N, providing critical data for structural health assessment. These results demonstrate that the proposed digital twin serves as a robust physical foundation for diagnostic systems, enabling the identification of contact-induced vibrational signatures that are essential for training prognostic algorithms. This approach facilitates the autonomous monitoring of critical rotating machinery in demanding industrial and subsea applications, supporting the transition toward active balancing and model-based vibration control strategies.

## 1. Introduction

The operational integrity of high-performance rotating machinery relies heavily on the integration of advanced materials capable of withstanding extreme pressures and corrosive environments. In this context, Polycrystalline Diamond (PCD) bearings have emerged as a pivotal technology, demanding the development of high-fidelity digital twins and sophisticated non-linear dynamic modelling to predict complex rotor–stator interactions. These validated models provide the essential physical foundation for both active model-based balancing and advanced prognostic strategies in critical industrial applications, such as remote offshore installations. While the fundamental breakthrough in reproducible diamond synthesis occurred in the mid-20th century, the subsequent decades have seen these material typologies transition from laboratory curiosities to essential industrial components [1,2,3,4]. This evolution is extensively documented in the literature, reflecting a maturation of manufacturing processes that now enables the deployment of synthetic diamond in the most demanding mechanical interfaces. The transition of PCD from a laboratory material to a critical engineering component has been extensively investigated through its tribological and mechanical performance in diverse operating environments. Early studies established the foundational wear-in behavior and load-carrying capacity of PCD thrust bearings, particularly for down-hole oil and gas drilling tools [5,6]. This research path has expanded into marine hydrokinetic (MHK) applications, where the material’s ability to operate in open-water lubrication regimes offers a significant advantage for renewable energy devices [7,8]. Recent experimental works have further characterized the abrasion resistance of sliding components in such hydrokinetic systems [9], while also exploring the potential of PCD in high-precision biomedical fields, such as hard-on-hard bearings for hip prostheses, highlighting its versatile stress distribution capabilities [10]. In water-lubricated conditions, the friction and wear behaviors of self-mated PCD pairs have shown superior performance compared to traditional air-lubricated environments. The influence of diamond grain size has been identified as a critical factor affecting both the tribological efficiency and the ultimate loading capacity of these friction pairs [11]. Moreover, the high-temperature stability of PCD under water-based drilling fluid environments remains a key focus for deep-well operations [12], supported by experimental measurements that quantify friction performance across varying operational parameters in oil drilling contexts [13]. As the complexity of turbomachinery increases, particularly in micro-applications and cryogenic pumps, the integration of specialized bearing designs has become essential to manage both static and dynamic loads. Recent studies have extensively investigated the performance of PDC bearings under varying drilling conditions [14], while innovative solutions such as multi-layer gas foil thrust bearings have been analyzed to determine their thermo-elasto-hydrodynamic behavior [15], static stiffness, and lift-off characteristics [16,17]. However, the deployment of these technologies in subsea environments, such as in Electric Submersible Pumps (ESPs), introduces significant rotordynamic challenges. Recent research has emphasized that ensuring system reliability requires a deep understanding of complex interactions, including the influence of housing–casing fluids on lateral rotordynamic stability [18], which is critical for maintaining operational integrity in demanding offshore operations. In order to address these challenges, the modern industrial landscape has increasingly adopted advanced optimization and data-driven monitoring strategies to enhance system resilience [19]. In the field of turbomachinery, techniques such as topology optimization for compressor blisks [20] and the dynamic analysis of additive manufacturing components [21] have demonstrated how high-fidelity numerical models can drive performance. Specifically, the design of bearing components has evolved through multi-objective evolutionary algorithms [22,23,24], aiming to ensure stability under variable operating conditions. This methodological shift is not confined to turbomachinery; similar paradigms in the automotive and railway sectors, ranging from prognostic health monitoring [25] to the development of physics-based digital twins [26,27], underscore the global necessity for intelligent fault diagnosis and fault severity assessment in rolling and sliding interfaces [28,29,30,31]. Accurate numerical representation of mechanical interactions is central to the success of these predictive architectures. The selection of an appropriate contact force model is a long-standing challenge in multibody system dynamics, as highlighted by extensive literature surveys on the evolution of contact modelling [32]. Research has progressed from classical Hertzian theory to advanced compliant contact force models [33], with recent reviews emphasizing the importance of continuous contact force formulations to capture the dissipative nature of impacts [34]. Specialized studies have further explored non-linear phenomena within multibody systems, proposing refined continuous models to handle the high-frequency dynamics of impact analysis [35,36]. However, the effective deployment of digital twins for high-performance PCD bearings is strictly dependent on the formulation of a validated non-linear framework capable of accurately replicating the stochastic nature of rub-impact events. This research work bridges the gap between material-level studies and system-level monitoring by proposing a calibrated multibody framework. The original contribution of this study lies in the integration of experimental modal analysis with transient contact simulations; specifically, it provides the physical foundation for the autonomous health monitoring of critical rotating machinery. By establishing these validated dynamic benchmarks, the study facilitates the transition from reactive to prognostic maintenance in demanding operational environments, such as remote offshore installations, while enabling the development of advanced frameworks for active model-based balancing and vibration control.

## 2. Experimental and Numerical Methodology

The research integrates experimental investigations with advanced numerical modelling to characterize the non-linear dynamics of PCD bearings. This methodology aims to establish a high-fidelity digital twin, providing the physical insights and vibrational benchmarks necessary to support the condition monitoring and the development of active model-based balancing strategies of critical rotating machinery in demanding operational environments.

### 2.1. Research Framework and Digital Twin Concept

The proposed framework is divided into three interconnected phases: experimental activity, numerical development, and model calibration/validation. The experimental phase is centered on a custom-designed test rig, where real-time monitoring of vibration accelerations, shaft orbits, and torque losses is performed during high-speed test to capture the onset of non-linear phenomena. This is complemented by structural characterization via ping tests, which provide the essential baseline for natural frequencies and damping to tune the subsequent structural Finite Element (FE) model. Concurrently, a high-fidelity digital twin is developed, starting with a 3D FE model in ANSYS 2021R2 to define the equivalent stiffness of the stator and supports, followed by a dedicated PCD contact model to derive the non-linear stiffness and damping parameters governing rub-impact interactions. These structural and tribological inputs are then integrated into a Multibody Dynamics environment within MATLAB-Simulink 2021, capable of simulating global dynamic behavior and predicting chaotic regime transitions. Once the calibration converges, the model is validated against independent data sets, serving as a physical foundation for the future implementation of active model-based balancing and vibration control. This framework provides a robust basis for feature extraction, identifying specific vibrational signatures in high-performance applications where high-fidelity simulations are essential for ensuring operational integrity and enabling autonomous, prognostic decision-making in demanding environments such as remote offshore installations.

### 2.2. PCD Test Rig Description

**Figure 1:**
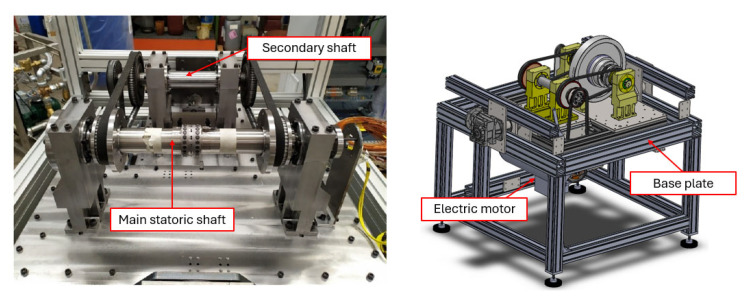
Overview of the PCD bearing test rig: comparison between the physical experimental apparatus (**left**) and the corresponding CAD digital model (**right**) [37].

The experimental campaign was conducted on a custom-built test rig designed to replicate the dynamic behavior of a single independent rotor within a complex turbomachinery system. The rig’s architecture, detailly described in [37] and shown in Figure 1, was specifically engineered to decouple the rotor’s dynamic response from the rest of the assembly while maintaining inertial properties representative of industrial subsea machines. A key feature of the system is its modularity, allowing for the easy variation in the rotational axis inclination and the testing of different PCD bearing configurations at speeds up to 6000 rpm.

The experimental campaign centered on a high-fidelity sensing suite designed to characterize the rotor–stator interaction and evaluate energy dissipation through the simultaneous acquisition of rotor displacements, stator vibrations, and torque absorption. The experimental matrix investigated three distinct PCD bearing configurations, primarily differentiated by their radial rotor–stator clearance: Baseline (50 µm), Low Clearance (40 µm), and High Clearance (200 µm). While the preliminary performance assessment and frictional behavior of these specific configurations are detailed in a previous study [37], the present work focuses on the non-linear dynamic characterization and the validation of a high-fidelity digital twin framework. This approach provides the physical foundation necessary for advanced monitoring and active balancing strategies. The following sections offer a detailed technical description of the test rig components and the specific sensor integration required to capture the dynamic parameters essential for model correlation.

### 2.3. Experimental Setup and Data Acquisition

**Figure 2:**
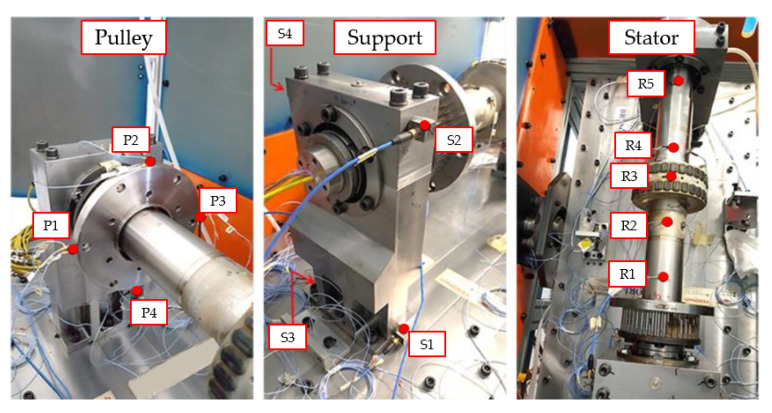
Instrumented test rig for experimental characterization: detailed view of the installed on the pulley, support, and stator components.

In order to characterize the dynamic behavior of the main statoric assembly, an extensive impact test (hammer test) was performed. The primary objective of this experimental campaign was to determine the modal parameters, specifically natural frequencies and damping ratios, within a 0–1500 Hz frequency range, with a particular focus on identifying the bending modes of the structure. As illustrated in Figure 2, the test rig was evaluated in a specific configuration comprising the two main supports, the stator axis, the pulley, and the PCD stator bearing. To ensure high-fidelity signal transmission, all accelerometers were mounted at the designated measurement points using beeswax. Prior to the execution of the tests, the entire measurement chain was rigorously verified using an accelerometer calibrator and a suspended rigid block of known mass to ensure sensitivity accuracy. The excitation was delivered at three strategic points using an instrumented hammer equipped with a hard tip to excite the full frequency range of interest. The dynamic response was captured via a multi-channel acquisition system consisting of twelve uniaxial and four triaxial accelerometers, distributed across seventeen measurement points. The complete technical specifications of the instrumentation employed are summarized in Table 1.

**Figure 3:**
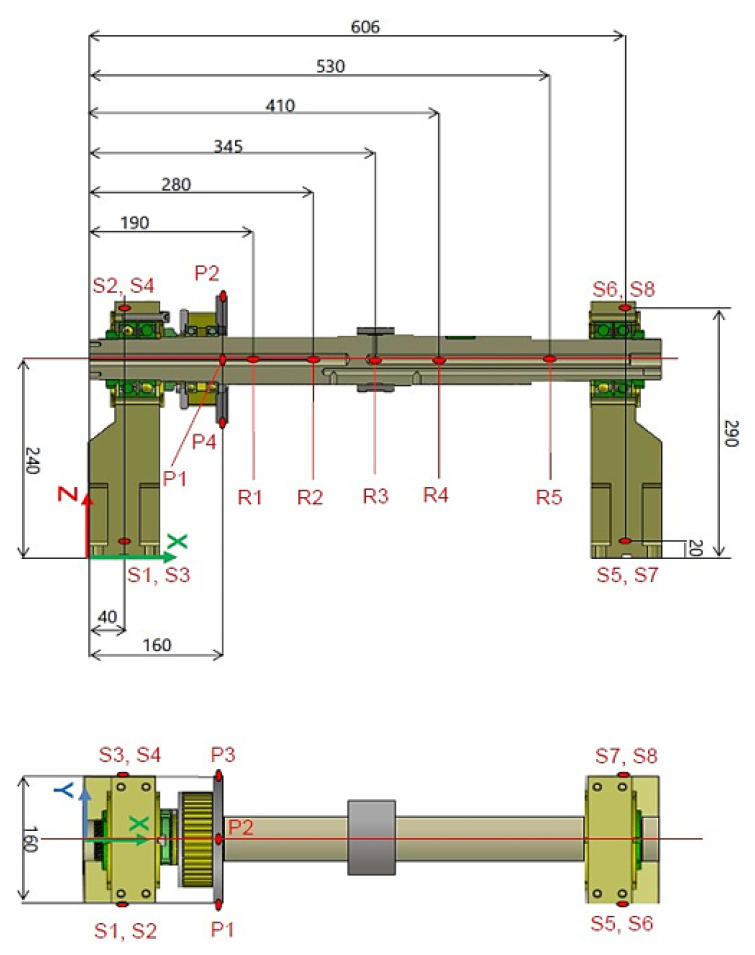
Dimensional layout and sensor placement: global coordinate system and spatial coordinates of the measurement points (all dimensions in mm).

**Figure 4:**
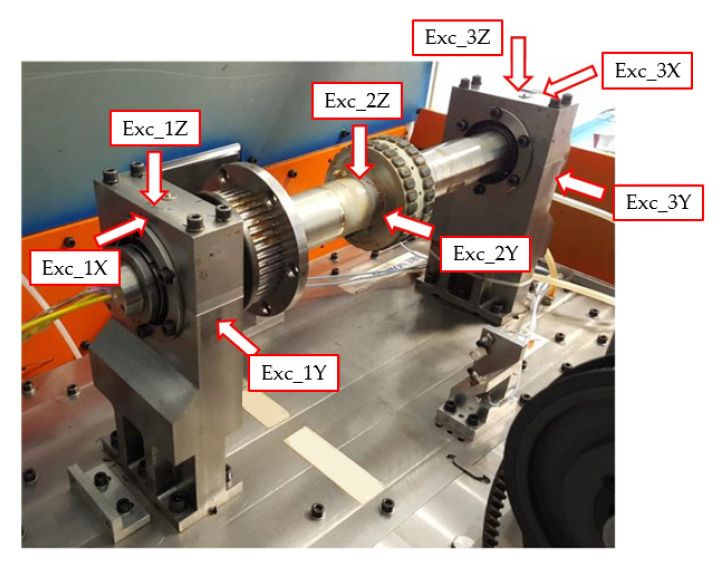
Impact testing configuration: identification of the excitation points and corresponding excitation directions for the experimental modal characterization.

Data acquisition and signal processing were performed using Simcenter TestLab (v. 18.2) with a bandwidth of 3200 Hz, applying an average of four signals for each measurement to ensure statistical consistency. The global reference system (refer to Figure 3) was defined with the *X*-axis aligned to the stator axial direction, the *Y*-axis along the horizontal radial direction, and the *Z*-axis in the vertical radial direction. The experimental campaign followed a roving accelerometer approach, where impulsive excitations were applied at three strategic locations (Figure 4): excitations 1 and 3 were delivered to the two main supports along all three axes (X, Y, Z), while excitation 2 was applied to the stator along the radial Y and Z directions. To minimize the influence of output noise, which is typical in impact testing, the Frequency Response Functions (FRFs) were calculated using the H1 estimator. Signal windowing was optimized by applying a transient window to the excitation and an exponential window to the response signals. Finally, the modal parameters, including natural frequencies and damping ratios, were extracted using the PolyMAX algorithm, ensuring a high-fidelity identification of the system’s dynamic properties. The system’s dynamic response was captured across seventeen strategic measurement points. To provide a comprehensive modal map, four points were located on each support (S1–S8) and four on the pulley (P1–P4), all utilizing triaxial accelerometers to capture 3D motion. Additionally, the stator was instrumented along five cross-sections: sections R1, R3, and R5 were dedicated to radial displacement monitoring (Y and Z directions), while sections R2 and R4 employed triaxial measurements to account for axial–radial coupling.

### 2.4. Digital Twin Formulation: Non-Linear Multibody Dynamics

The core of the numerical framework consists of a non-linear Multibody model developed in the MATLAB-Simulink^®^ environment, aimed at predicting the complex dynamic response of the PCD bearing test rig. The architecture of the model is centered on the interaction between two primary subsystems: a rigid rotor, representing the high-inertia disk, and a flexible statoric shaft. To ensure high-fidelity results, the shaft’s flexibility is modelled by integrating the structural parameters (stiffness and damping) previously identified through the experimental modal analysis and FE characterization. This dual-component approach allows for the accurate simulation of the rotor–stator coupling, providing a platform to investigate the non-linear forces arising within the PCD bearings and to predict potential regime transitions under high-speed operational conditions.

#### 2.4.1. Rotor Dynamics: Kinematics and Governing Equations

The dynamic response of the rotor is modeled by considering four primary Degrees of Freedom (DOFs). While the rotor maintains a constant spin speed Ω around its longitudinal axis (*z*-axis), the vibration dynamics are captured through two radial translations, x and y, and two tilting rotations, α and β, representing the nutation and precession around the transverse axes. This 4-DOF approach is essential to correctly characterize the gyroscopic coupling and the non-linear trajectories during rotor–stator contact. The vector of generalized coordinates is thus defined as:

$$q={\left[x,y,\alpha ,\beta \right]}^{T}$$

This formulation ensures that the model can accurately predict the shaft orbits and the stator interactions described in the experimental setup, focusing on the lateral-torsional decoupling typical of high-speed turbomachinery. The equations of motion are derived by accounting for gyroscopic effects, which are critical at the high operational speeds investigated in this work. The dynamic equilibrium of the system is expressed as:

$$M\ddot{q}+\left(G\Omega +C\right)\dot{q}+Kq=F_{unb}+F_{cont}$$

In this formulation:

- *M* is the mass matrix containing the rotor mass *m* and the transverse moment of inertia $I_{t}$;
- *G* is the gyroscopic matrix, which couples the tilting motions (α, β) through the polar inertia $I_{p}$ and the spin speed Ω;
- *K* and *C* represent the equivalent stiffness and damping matrices of the statoric assembly, where the support parameters have been calibrated using the experimental modal data (ping tests) and refined via 3D FE analysis;
- $F_{unb}$ denotes the synchronous excitation force vector resulting from the residual mass unbalance;
- $F_{cont}$ represents the non-linear contact force vector arising from the interaction between the rotor and the PCD bearings, which is triggered when the radial displacement exceeds the clearance, described in the next paragraph.

#### 2.4.2. Analytical Formulation of Rotor Rub-Impact Forces

The interaction between the rotor and the PCD bearing is governed by an impulse-based contact model, where the momentum of the disk is modified upon collision according to a defined coefficient of restitution. The contact forces are decomposed into normal and tangential components to accurately simulate rub-impact phenomena. The normal force magnitude, $F_{n}$, is activated only when the rotor displacement exceeds the radial clearance, c, resulting in a positive indentation δ:

$$F_{n}=K_{cont}\delta +C_{cont}\dot{\delta }$$

where δ is defined as the difference between the stator-rotor center distance and the radial clearance; $\dot{\delta }$ represents the indentation speed. The force vector acts along the normal unit vector n, defined by the line connecting the stator center (S) and the rotor center (R). Since the contact point P lies on this line, the vector (P-S) is parallel to (R-S), simplifying the directional definition of the force. The tangential force, $F_{t}$, arises from the friction at the PCD interface. Its magnitude is proportional to the normal force through a friction coefficient μ which can be modelled as a constant or as a function of the sliding velocity:

$$F_{t}=\mu F_{n}$$

**Figure 5:**
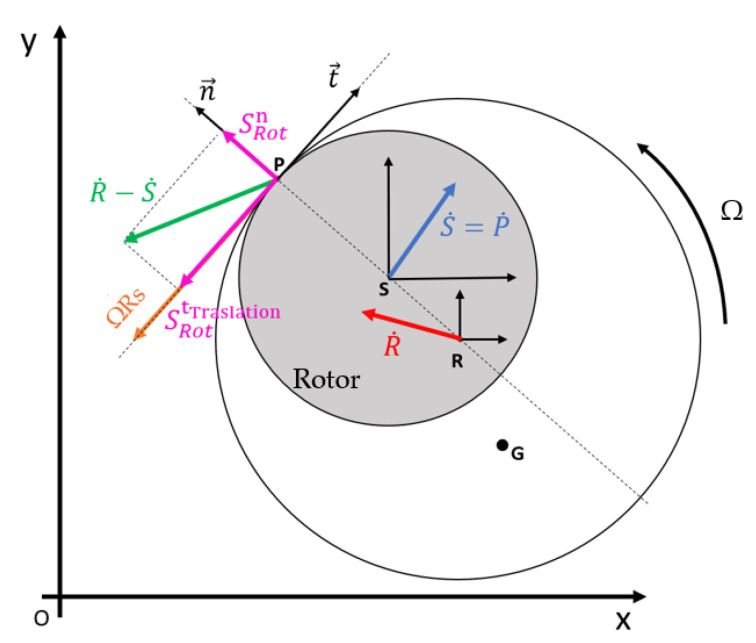
Kinematic scheme of the rotor–stator rub-impact: vector decomposition of normal and tangential velocities at the contact point P.

The orientation of the tangential force is determined by the relative sliding velocity between the rotor and stator at the contact point P. This tangential sliding, $v_{sl}$ is critical for describing the transition to whirl motion and is defined as:

$$v_{sl}=\left(v_{R}-v_{S}\right)\cdot \tau$$

where τ is the tangential unit vector. By evaluating the sliding components relative to the reference versors, the final vectorial form of the tangential force is established to account for the change in direction during complex orbital trajectories. The fundamental kinematic relationships and the resulting contact forces at the rotor–stator interface are graphically summarized in Figure 5, which illustrates the vector decomposition used to implement the non-linear rub-impact dynamics within the multibody framework.

#### 2.4.3. Stator Structural Modelling and 1D Finite Element Discretization

The stator is modeled as a flexible component to capture its structural influence on the overall system dynamics. Unlike the rotor, the stator is discretized using a 1D Finite Element (FE) model based on Timoshenko beam elements. This choice allows for the inclusion of shear deformation and rotary inertia, which are non-negligible for the statoric shaft geometry. Each element consists of two nodes with four DOFs per node (two translations and two rotations). The material properties assigned to the FE model correspond to structural steel (Young’s Modulus E = 210 GPa, Poisson’s ratio nu = 0.3, and density *ρ* = 7850 kg/m^3^). The global stiffness and mass matrices are assembled from the local element matrices, accounting for the specific cross-sectional variations and geometric properties of the shaft. The stator mesh incorporates several strategic nodes designed to accurately represent the physical interfaces and boundary conditions of the test rig. At the shaft ends, specific support nodes are defined to integrate the pedestal and roller bearings, which are modeled as equivalent spring-damper systems. The transmission system is represented by nodes corresponding to the pulley position; here, the belt’s preload and stiffness are applied through a unidirectional constraint that acts exclusively in the horizontal plane, ensuring that the belt cannot generate compression forces. Additionally, an instrumentation node is precisely positioned to coincide with the experimental accelerometer location, facilitating direct model validation. Finally, the model includes dedicated contact nodes where the non-linear rotor–stator interaction forces are applied, enabling the dynamic coupling between the flexible stator and the rotating assembly. The boundary conditions and external excitations include the support reactions, the belt load, and the contact forces. Specifically, the support pedestals and roller bearings are integrated as lumped parameters (K, C), calibrated to match the damping and stiffness identified in the experimental campaign. Crucially, the contact forces calculated for the rotor are applied to the stator nodes with equal magnitude and direction but opposite sense, ensuring the dynamic coupling of the two subsystems.

#### 2.4.4. Implementation of Stator-Pad Interaction Laws

**Figure 6:**
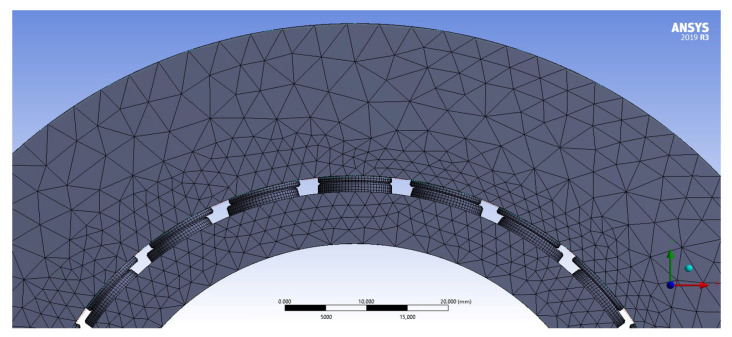
Finite element mesh used for the numerical characterization of the contact stiffness Look-Up Table (LUT).

The interaction between the rotor and the stator is decomposed into normal and tangential components to accurately evaluate the impact forces. The normal force magnitude is governed by a spring-damper model, with a specific focus on the contact stiffness (*K_cont_*). Due to the unique geometry of the PCD bearing surfaces, the stiffness is estimated using a cylinder-to-cylinder contact formulation based on Hertzian theory:

$$K_{cont}=\frac{\pi \cdot E_{eq}\cdot D_{b}}{2\left(1-\nu^{2}\right)}$$

where $D_{b}$ is the characteristic dimension of the contact zone (button diameter) and $E_{eq}$ is the equivalent Young’s modulus of the PCD interface. Given the high elastic modulus of Polycrystalline Diamond (E = 841 GPa, ν = 0.2), the contact stiffness reaches an order of magnitude of 10^9^ N/m. The finite element representation is shown in Figure 6.

**Figure 7:**
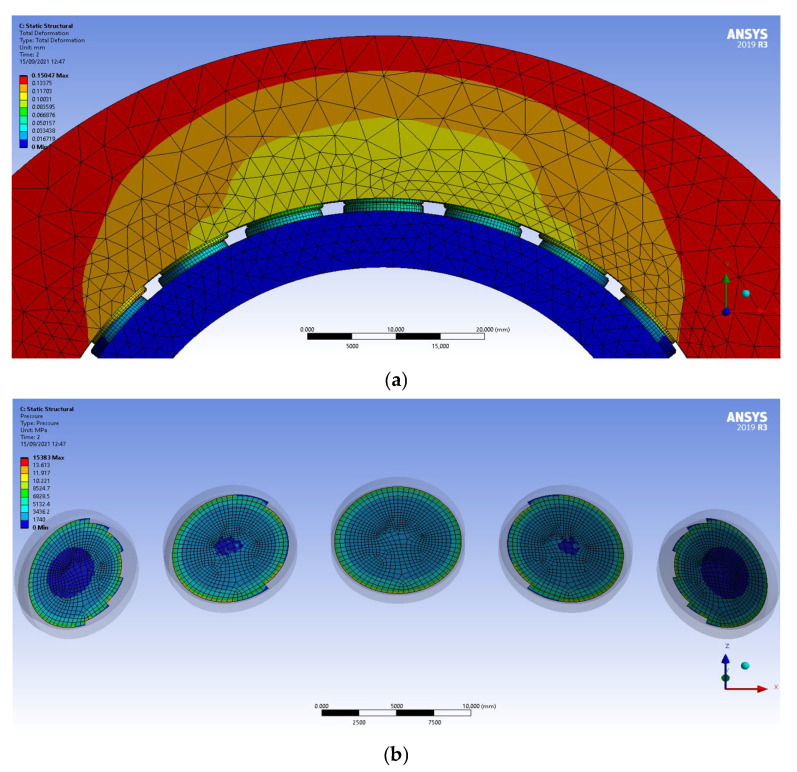
Results of the static structural FE analysis: (**a**) total displacement field of the assembly under contact loading, and (**b**) detailed contact pressure distribution on the PCD pads.

In this preliminary FEM phase, the stator axis was assumed to be rigid to isolate and evaluate the contact stiffness of the PCD interface. A fixed support boundary condition was applied to the internal surface of the male ring bearing. The radial clearance was then closed through a rigid transformation, such that the displacement applied to the external surface of the female ring bearing directly represented the indentation δ. The resulting displacement field is illustrated in Figure 7a, while Figure 7b presents the pressure distribution acting on the pads, providing the basis for the stiffness Look-Up Table used in the multibody environment.

## 3. Model Validation and Performance Analysis

This section presents the experimental validation of the numerical model and discusses the dynamic response of the system under different operational conditions. The analysis is structured in two main phases. First, a structural calibration of the 1D flexible stator model is performed by comparing the numerical natural frequencies with the experimental modal data obtained through ping tests. This step is fundamental to ensure that the support stiffness and damping parameters correctly replicate the test rig’s physical assembly. Subsequently, the non-linear dynamic response is investigated by comparing numerical simulations and experimental measurements in terms of rotor orbits and accelerometric signals. The study explores various test rig configurations, focusing on the influence of different rotational speeds and radial clearances. This systematic comparison aims to demonstrate the model’s capability to accurately predict the onset of rub-impact phenomena and the transition between different vibration regimes, providing a robust framework for the condition monitoring of PCD bearings.

### 3.1. Structural Correlation: Experimental Modal Analysis vs. FE Model

**Figure 8:**
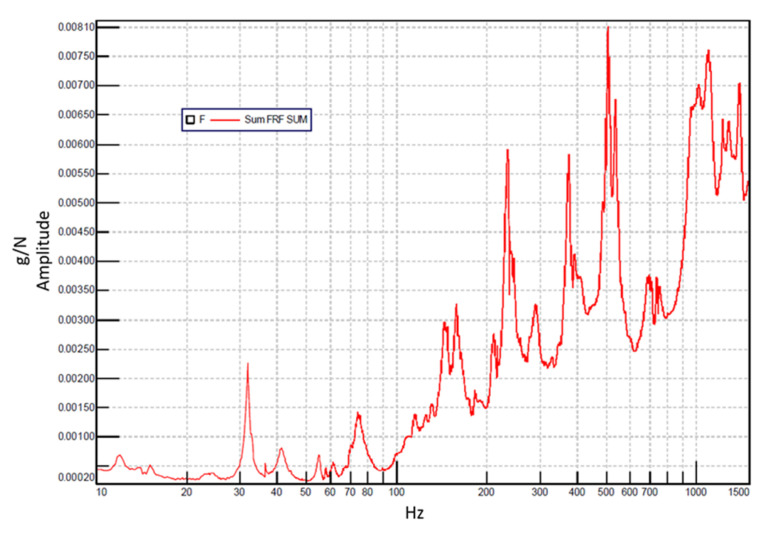
Sum FRF in 0–1500 Hz range.

The calibration process begins with the experimental modal analysis of the stator assembly. To ensure a high-quality excitation, the rule of thumb dictates that the force spectrum amplitude should not decay by more than 20 dB within the frequency range of interest. In this study, the impact excitation provided a remarkably flat spectrum, with a decay of less than 1 dB over the investigated range (0–1500 Hz), ensuring sufficient energy to excite all relevant structural modes. Frequency Response Functions (FRFs) were calculated for each measurement point across all excitation cycles. Figure 8 presents the SUM FRF, representing the average of the acquired signals. From this global response, stable poles were identified to extract the natural frequencies and the associated mode shapes. Furthermore, the experimental damping ratios were calculated for each mode, providing the necessary data to refine the damping matrix (C) used in the numerical model.

**Figure 9:**
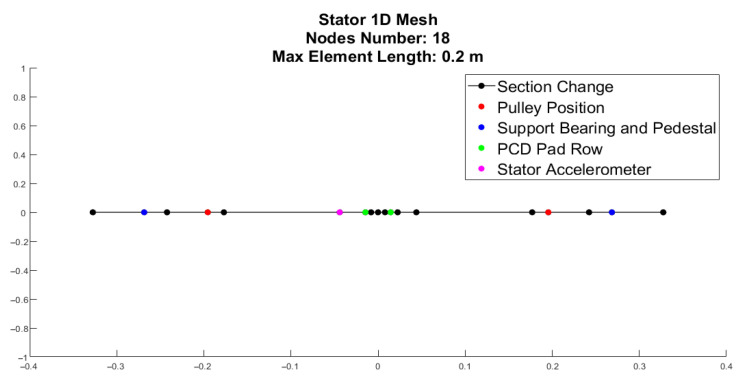
One-dimensional nodal discretization of the stator model, highlighting the strategic positioning of key components and experimental measurement points.

The calibration of the 1D FE stator model involves adjusting specific stiffness and inertial parameters to align the numerical eigenvalues with the bending modes identified during the impact (hammer) testing. The discretization used for this validation, shown in Figure 9, consists of a mesh with 42 nodes and a maximum element length of 0.02 m, providing sufficient resolution to capture the first four bending modes of the assembly.

The calibration focused on three categories of parameters:

1. Material and geometry, including the Young’s Modulus (E), density (ρ), and the masses of the PCD rings and transmission pulley were kept at their nominal design values;
2. Support stiffness, since the exact boundary conditions of the pedestals and roller bearings are difficult to characterize analytically, the translational ($k_{s}$) and rotational ($k_{\theta s}$) stiffness values were iteratively adjusted within physically plausible ranges to match the experimental natural frequencies;
3. Equivalent inertia, considering that it was observed that not all the physical mass or rotational inertia of the supports participates in the high-frequency vibration modes. Consequently, calibrated values, lower than the static measurements, were implemented for the support mass ($m_{support}$) and moments of inertia ($I_{s}$) to account for this effective participating mass.

The final set of parameters used for the structural model is summarized in Table 2.

**Figure 10:**
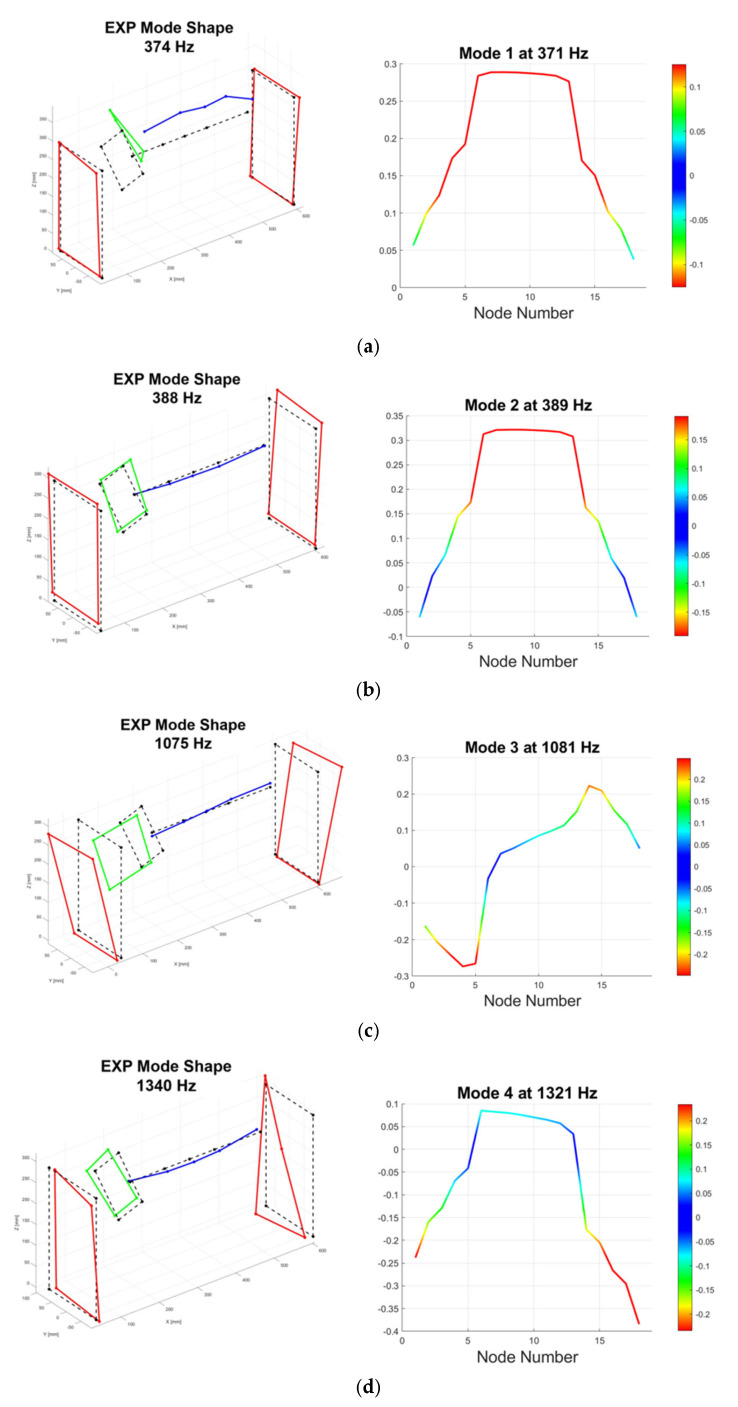
Comparison of experimental and numerical mode shapes for the stator assembly: (**a**) first mode at 374 Hz, (**b**) second mode at 388 Hz, (**c**) third mode at 1075 Hz, and (**d**) fourth mode at 1340 Hz, showing the high degree of correlation between the measured deformations and the model predictions.

The effectiveness of the calibration process is demonstrated by the close agreement between the experimental and numerical results. The structural dynamic behavior of the stator is characterized by four primary bending modes identified within the 0–1500 Hz range, demonstrating a strong correlation between the experimental data and the 1D FE model results illustrated in Figure 10. At lower frequencies, the system exhibits a high modal density where the first two modes occur in close proximity, 374 Hz and 388 Hz experimentally, both characterized by symmetric displacement profiles. Specifically, the first mode in Figure 10a shows maximum deflection at the PCD pad row, identifying this frequency as a critical threshold for the stability of rotor–stator engagement. This symmetric behavior transitions into a more complex response with the third mode at 1075 Hz, shown in Figure 10c, which introduces an antisymmetric displacement profile where the shaft exhibits a central nodal point with its two halves moving in opposite directions. The sequence culminates in a fourth bending state at 1340 Hz in Figure 10d, where the numerical model effectively captures the rapid displacement gradients near the shaft ends with a remarkably low error of approximately 1.4%. Overall, the precise alignment across these diverse vibrational states confirms that the calibrated stiffness and mass parameters provide a solid foundation for the subsequent non-linear transient simulations of contact events.

In order to conclude this phase of the study, the comparative analysis between the experimental measurements and the numerical predictions has been synthesized to provide a clear overview of the model’s accuracy. The specific values for the natural frequencies, along with the calculated percentage errors for each identified mode, are appropriately summarized in Table 3. This summary serves as a quantitative benchmark, confirming that the structural discretization and the subsequent parameter tuning effectively replicate the global dynamic behavior of the physical test rig.

### 3.2. Validation of Non-Linear Multibody Model

In order to validate the non-linear multibody model, the rotational speeds most representative of the machine’s operational range were investigated. Specifically, for the Baseline and Low Clearance vertical configurations, the analysis focused on rotational speeds of 3500, 4500, and 5500 rpm. In contrast, the High Clearance configuration was validated at 1500, 2500, and 3500 rpm, as higher speeds were restricted by the structural stability limits of the test bench. For the sake of brevity, this section reports the results corresponding to the highest validated speed for each configuration. The model validation is based on a comprehensive comparison of diverse physical quantities, including rotor orbits, accelerometric signals, and torque absorption. To ensure the numerical environment accurately replicates the experimental behavior, the model was calibrated by tuning the damping coefficients of the structural supports, the metal bellows, and the PCD contact interface, as well as the residual unbalance of the rotor assembly. Finally, the analysis presents the mean values of the contact forces across all configurations. These results are fundamental to characterizing the mechanical interaction between the rotor and the statoric components, providing a quantitative measure of the impact severity during rub-impact events.

#### 3.2.1. Shaft Orbital Response

**Figure 11:**
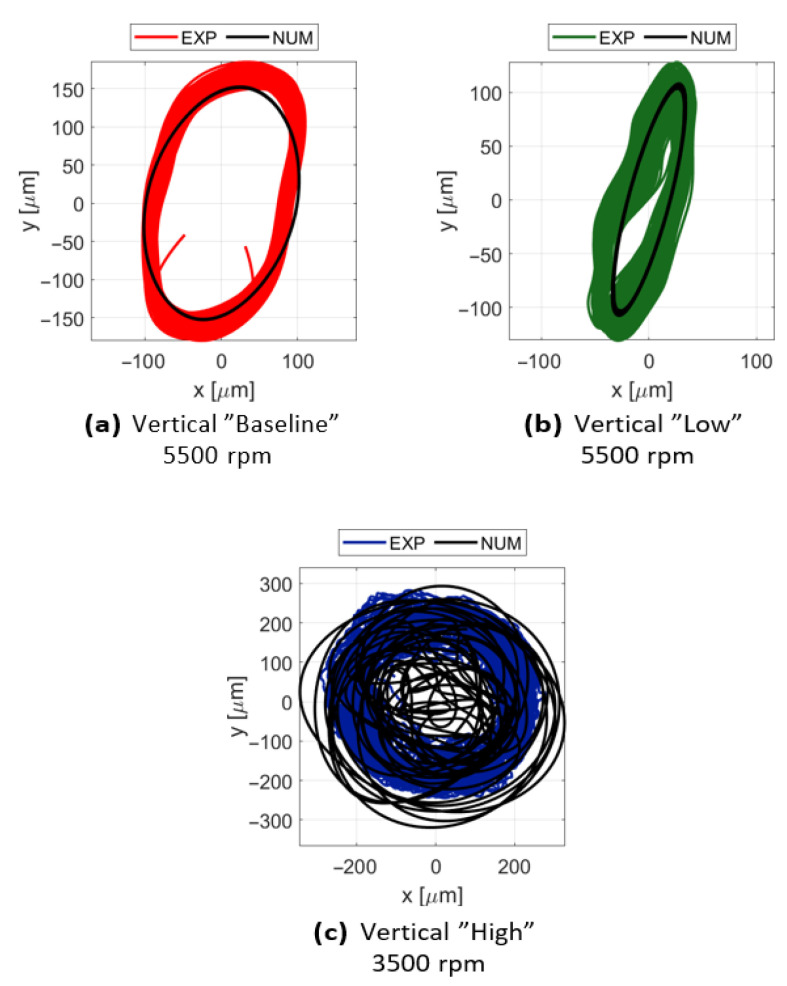
Validation of the shaft orbits: comparison between experimental (EXP) and numerical (NUM) trajectories under different clearance conditions and rotational speeds, illustrating the model’s ability to replicate both stable synchronous whirl and non-linear chaotic responses.

The capability of the non-linear multibody model to replicate the experimental rotor response is evidenced by the orbit comparisons across different bench configurations. As illustrated in Figure 11, the numerical model successfully captures the fundamental morphology of the orbits for all tested clearances. In the “Baseline” configuration (Figure 11a), the model shows excellent agreement with the experimental data, tracing a stable and repeatable path at 5500 rpm. However, as the system transitions toward the “Low” and “High” clearance configurations (Figure 11b,c), the experimental data reveal a significantly more chaotic dynamic behavior, characterized by a broader dispersion of the rotor trajectories. This stochastic nature is particularly evident in the “High” configuration at 3500 rpm, where the increased radial gap facilitates complex rub-impact sequences. Numerically, the model is able to reproduce this disordered regime for the “High” configuration, effectively simulating the non-linear interactions and the scattering of the orbits. This orbital dispersion serves as a practical metric for identifying the transition to non-periodic regimes, where the sensitive dependence on contact initial conditions leads to the observed chaotic-like trajectories. While the “Low” configuration’s numerical orbit remains more contained than the experimental one, the model still correctly identifies the primary axis of vibration and the onset of contact-induced disturbances.

#### 3.2.2. Stator Vibrational Analysis

**Figure 12:**
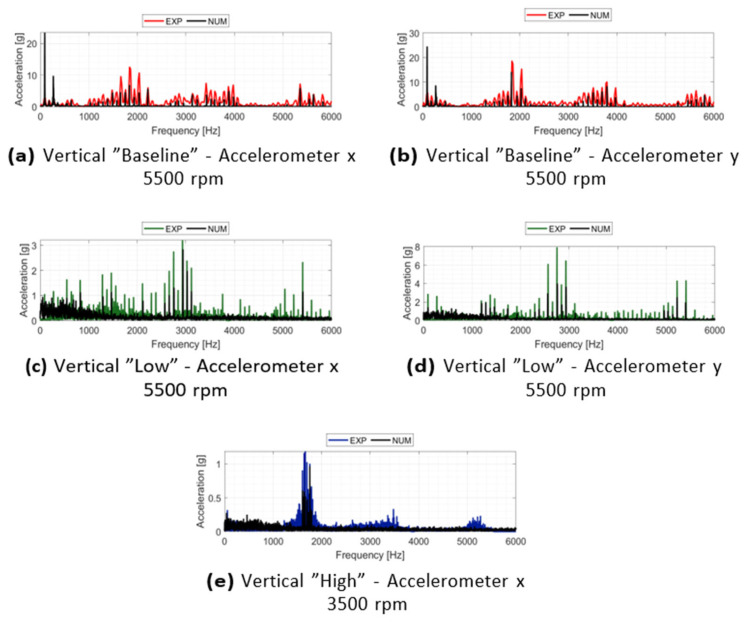
Spectral validation of the stator vibrations: comparison between experimental and numerical FFTs across different acceleration channels and operating conditions, showing the accurate replication of the high-frequency vibro-acoustic signatures.

The capability of the non-linear multibody model to replicate the vibration spectra is evaluated through the Fast Fourier Transform (FFT) of the acceleration signals. Figure 12 illustrates the comparison between experimental and numerical results for the “Baseline” and “Low” configurations at 5500 rpm, and the “High” configuration at 3500 rpm. The analysis includes both x and y directions, with the exception of the “High” configuration where data is limited to the *x*-axis due to a sensor failure during the experimental campaign. In all investigated cases, the model demonstrates a good capacity to reproduce the primary spectral features, specifically capturing the supersynchronous harmonics related to the number of pads. For the “Baseline” configuration shown in Figure 12a,b, a noticeable discrepancy is observed at the synchronous frequency (1×), where the numerical model predicts higher acceleration amplitudes than those recorded experimentally. This difference may be attributed to a slight overestimation of the residual unbalance or an underestimation of the damping provided by the metal bellows in the numerical environment. As the system transitions to the “Low” and “High” configurations (Figure 12c–e), the increased complexity of the rub-impact events leads to a more stochastic spectral distribution. While the numerical model effectively identifies the regions of highest energy density and the characteristic frequencies of the contact interface, a degree of discrepancy remains in the baseline noise level and peak resolution. These differences are consistent with the more chaotic dynamic behavior observed in the orbit analysis, highlighting the sensitivity of the system to the non-linearities of the clearance and the contact stiffness.

#### 3.2.3. Contact-Induced Torque Analysis

**Figure 13:**
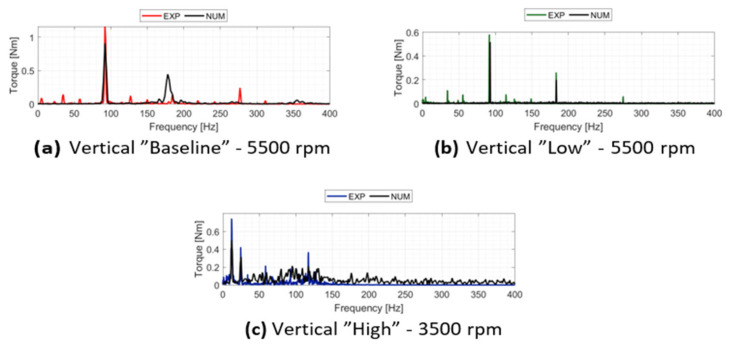
Validation of the torque spectra: comparison between experimental and numerical FFTs of the driving torque, highlighting the model’s precision in replicating the harmonic components induced by the rub-impact forces.

The final step of the multibody model validation involves the analysis of torque absorption in the frequency domain. As illustrated in Figure 13, a high level of correlation was achieved across all tested configurations, confirming the model’s ability to accurately estimate the energy dissipation associated with rotor–stator interactions. In the “Baseline” and “Low” configurations (Figure 13a,b), the dominant synchronous peaks are correctly identified. The only notable discrepancy appears in the “Baseline” case, where the numerical model predicts a 2× synchronous peak that is less pronounced in the experimental data. This localized difference is likely attributable to subtle variations in the rotor’s whirling motion or the specific alignment of the coupling system in the numerical environment. Furthermore, the torque spectrum for the “High” clearance configuration at 3500 rpm (Figure 13c) clearly reflects the chaotic dynamic behavior previously identified in the orbit and acceleration analyses. The broad-band frequency content and the distribution of energy across the spectrum are well-replicated by the model, demonstrating its robustness in simulating high-clearance regimes where rub-impact phenomena become the primary driver of the system’s torque response.

#### 3.2.4. Numerical Estimation of Internal Contact Forces

**Figure 14:**
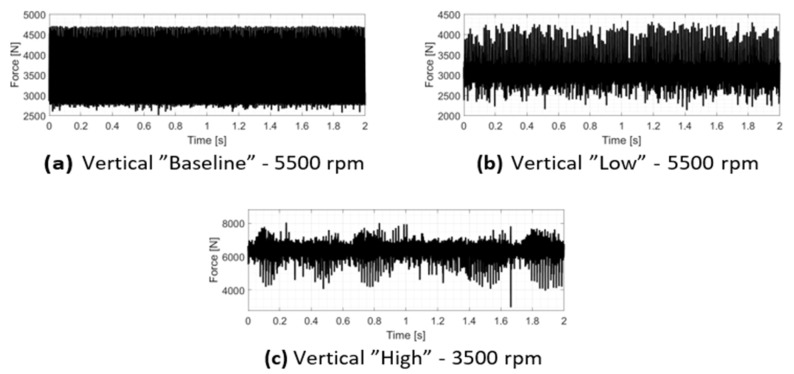
Time-domain estimation of the contact forces: numerical reconstruction of the impulsive loads at the rotor–stator interface for the three investigated operating conditions.

The temporal evolution of the contact forces for the three investigated configurations is presented in Figure 14. The results clearly illustrate the transition from periodic to stochastic interaction as the system clearance increases. In the “Baseline” configuration (Figure 14a), the force profile exhibits a remarkably regular and periodic behavior, consistent with the stable orbits previously discussed. In contrast, the “Low” and “High” clearance configurations (Figure 14b,c) show a significant increase in chaotic behavior, characterized by highly irregular force spikes and a less predictable distribution of impact events over time.

**Figure 15:**
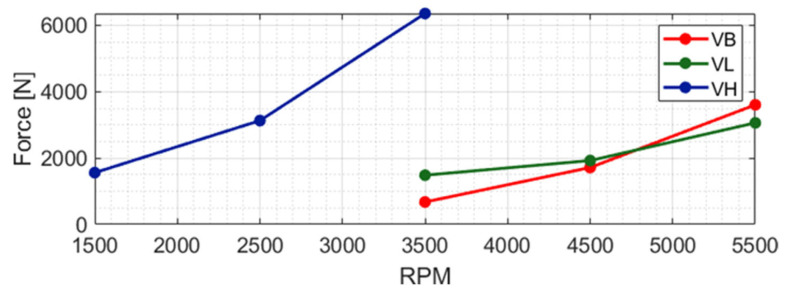
Evolution of the mean contact force as a function of rotational speed for the different clearance configurations (VB, VL, VH), highlighting the sensitivity of the impulsive loads to operating parameters.

The quantitative impact of these dynamics is further synthesized in Figure 15, which illustrates the trend of the average contact force as a function of rotational speed. The “High” clearance configuration (VH) stands out, generating substantially higher contact forces, reaching peaks significantly above the other cases, due to the increased radial gap that allows for more energetic and violent impacts between the rotor and stator. Conversely, the “Baseline” (VB) and “Low” (VL) configurations exhibit much lower average forces with a similar upward trend as rotational speed increases. Interestingly, the “Baseline” configuration maintains slightly lower force values up to approximately 4500 rpm; beyond this threshold, the “Low” configuration generates a lower average force, suggesting a shift in the contact regime or a change in the dominant vibration mode at higher speeds. These findings are instrumental in understanding the structural load requirements and the wear potential for the statoric components under realistic operating conditions.

## 4. Conclusions

The research presented in this work establishes a robust framework for the experimental characterization and non-linear dynamic modelling of PCD bearings, providing a validated digital twin approach essential for the condition monitoring and reliability of critical rotating machinery. The main contribution lies in the development of a validated analytical bridge between structural modal properties and non-linear rub-impact dynamics. The systematic calibration of the stator’s structural parameters enabled the development of a high-fidelity 1D FE model, which demonstrated a remarkable predictive capability by matching the first four experimental bending modes with a maximum frequency error of only 1.4% at 1321 Hz. This structural accuracy proved fundamental for the subsequent non-linear multibody validation, where the model successfully transitioned from reproducing stable, periodic orbits in baseline conditions at 5500 rpm to capturing the highly stochastic and chaotic trajectories characteristic of high-clearance configurations at 3500 rpm. The frequency-domain analysis further confirmed the model’s precision in identifying supersynchronous harmonics tied to the pad geometry, while the contact force investigation provided a quantitative measure of impact severity, revealing mean loads exceeding 6000 N in the most critical clearance regimes. Future developments will focus on integrating these validated contact dynamics with wear degradation laws to estimate the remaining useful life of the bearings. By correlating the observed chaotic vibrational signatures with long-term structural health, this modelling approach serves as a comprehensive foundation for the development of active model-based balancing and vibration control strategies. Consequently, the proposed digital twin successfully supports the implementation of advanced prognostic algorithms for critical rotating machinery in demanding environments, such as remote offshore installations.

## Figures and Tables

**Table 1:** Specifications of the instrumentation employed for the Experimental Modal Analysis (EMA).

Description	Quantity	Producer	Type
Accelerometer	12	PCB	352C23
	4	PCB	356A15
Accelerometer Calibrator	1	Bruel&Kjaer	4294
Instrumented Hammer	1	PCB	086C04
Data Acquisition System	1	LMS	SCR05
Laptop	1	DELL	PrecisionM4800

**Table 2:** Summary of structural parameters and results of the modal calibration process (Note: Values marked with ‘-’ were not analytically predetermined or available from manufacturer specifications; these parameters were uniquely identified through the experimental-numerical calibration procedure).

Model Parameter	Symbol	Real Value	Calibrated Value
Young Modulus	*E* [GPa]	210	210
Density	*ρ* [kg/m^3^]	7850	7850
PCD bearing mass	*m_PCD_* [kg]	3.103	3.103
Pulley mass	*mpulley* [kg]	3.04	3.04
Pulley inertia moments	$I_{x}$*^p^* [kgm^2^]	0.0047	0.0047
	$I_{y}$*^p^* [kgm^2^]	0.0047	0.0047
Support translation stiffness	$k_{x}$*^s^* [N/m]	-	1.5 × 10^8^
	$k_{y}$*^s^* [N/m]	-	5.5 × 10^8^
Support rotation stiffness	$k_{\theta }$*^s^* [Nm/rad]	-	3.8 × 10^5^
	$k_{\psi }$*^s^* [Nm/rad]	-	9 × 10^5^
Support mass	*msupport* [kg]	18	6
Supports inertia moments	$I_{x}$*^s^* [kgm^2^]	0.3189	0.3189 × 10^−2^
	$I_{y}$*^s^* [kgm^2^]	0.0444	0.0444 × 10^−2^

**Table 3:** Comparison between experimental and numerical natural frequencies after structural calibration.

Mode Number	Experimental [Hz]	Numerical [Hz]	Error [%]
1	374	371	1.0
2	388	389	0.2
3	1075	1081	0.6
4	1340	1321	1.4

## Data Availability

Data are contained within the article.
